# Bulimia nervosa as a risk factor for voice disorders - literature review

**DOI:** 10.1016/S1808-8694(15)30581-4

**Published:** 2015-10-19

**Authors:** Patricia Balata, Viviane Colares, Katia Petribu, Mariana de Carvalho Leal

**Affiliations:** 1 Specialist, Master's degree student, Faculty Member.; 2 Doctor, Adjunct Professor of Odontopediatrics, UFPE and UPE.; 3 Doctor, Adjunct Professor, UPE.; 4 Doctor in Science, USP Medical School, Otorhinolaryngologist at the Agamenon Magalhaes Hospital and the Instituto Real Otorrino (ENT Royal Institute) of the Portuguese Hospital, PE. Pernambuco University - Pernambuco Dentistry School.

**Keywords:** bulimia, voice disorders

## Abstract

Bulimia nervosa (BN) is a type of feeding disorder that starts in adolescence and presents a variety of symptoms, recurrent vomiting in the oral cavity that may reach down to the larynx - similarly to gastro-esophageal reflux, causing laryngeal and voice disorder alterations.

**Aim:**

These studies aimed at surveying the literature and investigate the studies that considered BN a risk factor for voice disorders.

**Results:**

of the ninety three papers we found, twenty-three were used as a basis for this review, among them, only three discuss BN as an etiology factor associated with voice changes in adult women, and we did not find any paper associating this with bulimic teenagers.

**Conclusion:**

It is necessary to observe laryngeal and vocal signs and symptoms associated with BN, especially in teenagers whose voices are going through a period of change. Keywords: bulimia, voice disorders

## INTRODUCTION

Nervous bulimia (NB) is a specific, invariably chronic type of eating disorder that usually begins in late adolescence.^1^

Gerald Russel first described this condition in 1979. Its main feature is the rapid and compulsive intake of large amounts of food, followed by the use of purgative methods such as provoked vomiting and the use of laxatives and diuretics, after which patients go through non-purgative periods that include fasting and inanition due to fear of gaining weight.^1^, ^3^

There have been few studies aimed at the adolescent population, possibly because only adults with this condition seek specific therapy for NB. On the other hand, there have been more studies on this subject, probably because of the incidence of this disease. The etiology of this disorder is related to various associated social, psychological and biological factors.^3^

The excessive value given to slim female bodies appears to be linked with an increased occurrence of eating disorders such as anorexia and nervous bulimia.2 Such cultural pressure, the media, and the collective imaginary encourage women in particular to go through sacrifices to attain an idealized body.^3^

Therapy for bulimic patients is multidisciplinary, involving mostly physicians, psychologists and nutrition specialists, given the complex factors that compose this disease. Best results tend to occur when early interventions are made in adolescence, which avoids the chronic and immutable states of eating disorders.^4^

Ninety-five percent of bulimic patients self-induce vomiting to minimize the anxiety that results from excessive eating (hyperphagia).^1^, ^16^

As a result of vomiting, the mouth may present findings such as xerostomia, oral mucosa irritation, dental sensitivity to temperature changes, radicular caries, among others.^5^, ^6^ Dental surgeons who observe these signs should investigate the presence of NB in their patients.

Vomiting and gastroesophageal reflux (GERD) may also affect adjacent structures to the esophagus, such as the upper aerodigestive tract, specifically the larynx.^9^, ^10^

Phlegm and chronic coughing that result from GERD-induced laryngeal irritation may result in laryngitis and dysphonia or voice disorders.^9^, ^10^

The presence of organic-type dysphonia in women with NB has been reported in studies that include clinical cases of patients complaining of hoarseness and voz grave, in whom laryngeal alterations are similar to those found in GERD patients.^11^

Another factor is important in adolescence, other than the physiological changes that occur in this age group as a result of hormone discharges, namely the teenager's voice. This period is named voice change, during which the voice fluctuates and changes tone.^14^

Considering NB as a risk factor for voice and laryngeal changes, the purpose of this paper was to investigate voice complications in individuals with NB, based on a review of the literature.

## METHOD

A review of the literature was done based on a survey of LILACS and MEDLINE databases, which are linked to the BIREME virtual library (http://bireme.br). The keywords “bulimia”, “adolescent” and “complications” were used. Citations with summaries were chosen to limit the topic, for the period between 1995 and 2006, in English and Portuguese.

Twenty-three papers were selected for this study. Books on the topic of this study, information collected from the Pan-American Health Organization (PAHO) (http://www.opas.org.br), from the Specialized Outpatient Eating Disorders Unit (Ambulin), Psychiatry Institute, Sao Paulo University Clinical Hospital (HC/USP) (http://www.ambulim.org.br), and from the Mental Health site (http://www.mentalhealth.com) were also used in this investigation.

The keyword “voice disorders” was added to find papers relating bulimia and voice disorders; in this case, only three papers not including adolescents were found, showing the paucity of papers on the interface voice disorders and bulimia.

### Bulimia and its complications

Galen, a Greek physician and philosopher born in 130 a.D., described the bulimic behavior as “kynos orexia” or canine hunger, and considered it as a consequence of an abnormal state of mind. The term bulimia appeared as a curiosity in medical dictionaries of the 17th and 19th centuries. The condition was described as bulimarexia in the 1970s until being defined as bulimia in the 1980s; the term nervous bulimia was finally adopted. The word originated from the Greek bous (bull) and limos (hunger).^1^, ^22^

NB is more common than nervous anorexia (NA), but is less easily detected; it may present initially as a major effort by a patient in controlling his or her weight. Its prognosis is better than that of NA, but the complications of NB may result in profound sequelae for patients, and may even be lethal.^18^

The clinical signs of NB are varied and may be severe; these signs include metabolic and hydroelectrolytic alterations, such as dehydration, hypocalemia, hyponatremia, hypomagnesemia, and metabolic alkalosis, all of which may be found in 50% of cases. These manifestations result from vomiting, fasting and laxatives.^1^

Gastrointestinal and oral complications are common, followed by electrolytic and endocrine alterations. Some patients develop severe conditions that require hospitalization and/or surgery on the organs that are affected most, such as the esophagus, the stomach and the intestines, in which perforation may occur. Chronic patients may also present heart disorders and brain dysfunction.^23^, ^24^

Oral alterations such as tongue lesions, increased parotids and tonsils, and dental erosion are all closely related with gastric acid regurgitation in NB or GERD patients.^25^, ^26^

Besides the inherent signs and symptoms of NB, there are three other important and visible manifestations that may be present: one is Russel's sign, which is a skin lesion caused by introducing the hand into the mouth to induce vomiting; the second sign is the “full moon” face, which is caused by salivary gland hypertrophy, mostly the parotids; and finally, there is dental erosion caused by gastric juice acidity contacting the mouth upon vomiting.^1^

Self-induced vomiting is a diagnostic criterion for NB. Vomiting may be provoked by hand or by introducing foreign bodies such as a plastic fork, which was reported in a case where this objects was swallowed into the hypopharynx, resulting in airway obstruction and dysphagia.^27^

Clinical manifestations are proportional to the level, quantity and frequency of gastric regurgitation, as well as other conditions such as esophagitis, gastritis, peptic ulcers and duodenal ulcers.^6^, ^7^

Swallowing is a function of the stomatognathic system that may be altered in NB. This disease may affect swallowing during the oropharyngeal phase, and may also interfere with taste, besides causing tissue injury. Taste is altered because gustatory receptors may be found in the palate, which is affected by acid vomiting.^28^, ^29^

Gastric content reflux may be physiological or pathological. The former is short-lived and occurs naturally after a meal. The latter is defined as reflux at a frequency, duration, intensity of events and quality of esophageal acidity that exceed physiological reflux criteria. These patients are said to have gastroesophageal reflux disease (GERD).^9^

Many papers in otorhinolaryngology, pediatrics and pneumology have been published because of GERD, which causes symptoms such as pharyngeal irritation, hoarseness, coughing, dispnea, thoracic pain, pneumonia, halitosis, retrosternal pyrosis, increased intra-abdominal pressure, odynophagia, dysphagia, a lump in the throat feeling, laryngeal spasm, and post-nasal aspiration.^8^, ^9^

### The bulimic individual

PAHO has reported that in the next decade the number of adolescents and youths will increase unprecedently worldwide. According to PAHO, this group is exposed to many risks and has various opportunities for leading their lives. About 70% of adult early mortality occurs in adolescence. Every year 1.4 million teenagers lose their lives due to intentional action, suicide and violence.^17^

NB manifests in adolescents as compulsive behavior strongly associated with personality disorders, anxiety, obsessive-compulsive disorder, depression, humor disorders, and use or dependency on substances such as alcohol and other stimulants.^3^

From a psychological perspective, there is a strong relation between eating disorders, such as NB and NA, and depression. Investigating the time sequence in which comorbid disorders manifest across life is an interesting life of research that has provided important insights. Stice et al.^19^ examined 496 female adolescents using multivariate analysis to investigate comorbidity between bulimia, depression and substance abuse. These authors found that depression was a predictive factor for bulimia, but not for substance abuse, while substance abuse preceded depression, but not NB. They concluded that there is comorbidity because certain disorders are risk factors for other disturbances.

In contrast, another study found that identifying and treating conditions such as anxiety disorders could improve the social adaptation of bulimic patients, as well as the underlying psychopathological entity, which highlights the need for early diagnosis and therapy in such cases.^20^

Although eating disorders are significantly more frequent in women, a Brazilian study of school children and teenagers aged between seven and 19 years found a high prevalence in males, revealing that, in recent years, more male individuals are developing eating disorders.^2^

These adolescents commonly abuse of laxatives and diuretic tablets for reducing weight. Induction of vomiting was the most frequently used method in adolescents with a possible diagnosis of BN.^2^

### Voice and bulimia

One of the physiological changes that adolescents undergo is voice change, which may be rapid, lasting not more than six months, and which is part of the development of secondary sexual characteristics.^15^

Voice change, also named transitional dysphonia or altered voice change, is most evident in male adolescents, resulting from increased hormone levels.^21^, ^14^ The pitch of a boy's voice becomes deeper (masculinized) as the adolescent grows into adulthood, becoming an important aspect of personality.^15^

Although NB usually initiates in adolescence or early adulthood, studies on NB have investigated mostly adult samples, possibly because medical help tends to be sought at this time.^3^

A North-American study of eight bulimic singers that also had GERD, aged between 24 and 34 years, revealed that they complained of hoarseness, laryngeal pain, voice fatigue, phlegm, lower-pitched voice, recurring voice loss and ardor. Laryngeal findings in this group were posterior edema, posterior commissure hypertrophy, ventricular obliteration, polypoid-type degeneration and teleangiectasia.^12^

Another case-report study that also related voice and laryngeal disorders with NB in a 29-year-old singer suggested that the clinical findings in this bulimic patient were similar to those found in GERD patients. The author, therefore, alerted healthcare professionals about the need to take bulimia into account as one of the causes of clinical pictures similar to GERD.^13^

NB patients may present upper aerodigestive tract complaints, requiring otorhinolaryngologists or other specialists to understand this disease for a correct diagnosis and therapy.^30^

NB with voice disorders may require speech therapy in addition to its usual treatment.^16^

## DISCUSSION

None of the papers that we reviewed related bulimia with voice disorders in adolescents. Three studies of female adults reported a close relation between NB and voice disorders, suggesting that NB might be a risk factor for the latter outcome.^11^, ^12^, ^13^

The literature that was reviewed in this study underlines the possible physical and psychological severity of NB, which evidently requires an effort to understand the etiology and pathophysiology of this condition.

Vomiting is a regurgitation-type event in which gastric content flows towards the mouth; this causes many symptoms in bulimic individuals, such as dental erosion, altered taste and swallowing disorders.^5^, ^6^, ^7^, ^23^, ^25^, ^27^, ^28^ It is not incongruous to think that acid gastric content might enter and injure the larynx, causing voice problems.

By analogy, purgative manifestations in NB are similar to those of GERD, which is a disease in which dysphonia is one of its main symptoms. Voice symptoms are usually associated with organic alterations involving the larynx and affecting phonation, such as edema, polyps and others. Interventions are clearly needed to avoid further laryngeal dysfunction.

Voice disorders in conditions such as NB are classified as Psychiatric Dysphonias,^16^ given the significant psychic component in the etiology and maintenance of this disease.

Voice has significant psychosocial importance; human beings use voice to express their thoughts, which in turn are enriched by the expressive voicing abilities.

As the voice of adolescents is susceptible to hormonal influences, and as bulimic disease starts in this age group, voice disorders should not be neglected in teenagers; it should be investigated regardless of the magnitude of signs and symptoms and clinical findings that deserve priority in bulimic individuals.

## FINAL COMMENTS

Although still incipiently, the indexed literature suggests that professionals involved in voice studies, such as speech therapists and otorhinolaryngologists, should pay attention to any signs relating voice disorders with NB.

Even though GERD and NB may present similar symptoms, the severity of bulimia requires more care in investigating the etiology of laryngeal and voice diseases.

The contribution of this type of investigation, which should begin with a clinical history, is essential for minimizing the complications of NB; thus, adolescents and adults with voice disorders should be investigated in greater detail.
